# Dual function of EDTA with silver nanoparticles for root canal treatment–A novel modification

**DOI:** 10.1371/journal.pone.0190866

**Published:** 2018-01-18

**Authors:** Juan M. Martinez-Andrade, Miguel Avalos-Borja, Alfredo R. Vilchis-Nestor, Luis O. Sanchez-Vargas, Ernestina Castro-Longoria

**Affiliations:** 1 Departamento de Microbiología, Centro de Investigación Científica y de Educación Superior de Ensenada (CICESE), Ensenada, Baja California, México; 2 Laboratorio Nacional de Nanociencias y Nanotecnología, Instituto Potosino de Investigación Científica y Tecnológica (IPICyT), San Luis Potosí, San Luis Potosí, México; 3 Centro Conjunto de Investigación en Química Sustentable, Universidad Autónoma del Estado de México y Universidad Nacional Autónoma de México (UAEM-UNAM), Estado de México, México; 4 Facultad de Estomatología, Universidad Autónoma de San Luis Potosí (UASLP), San Luis Potosí, San Luis Potosí, México; VIT University, INDIA

## Abstract

The chelating and antimicrobial capacity of a novel modification of 17% EDTA with silver nanoparticles (AgNPs) (EDTA-AgNPs) was evaluated *in-vitro* for root canal treatment (RCT). The EDTA-AgNPs solution was characterized by UV-Vis spectroscopy, ζ-potential and high-resolution transmission electron microscopy (HRTEM). Antimicrobial capacity was evaluated against *Candida albicans* and *Staphylococcus aureus* in planktonic and biofilm cells by broth macrodilution (24 h) and XTT assays, (1, 10 and 30 min) respectively. The chelating capacity of EDTA-AgNPs was assessed indirectly (smear layer removal) and directly (demineralizing effect) in bovine dentin at two silver concentrations, 16 and 512 μg/ml at 1 and 10 minutes of exposure time. Smear layer removal was evaluated by atomic force microscopy (AFM) and scanning electron microscopy (SEM). The demineralizing effect was determined by atomic absorption spectroscopy (AAS), microhardness test (MH) and X-ray diffractometer (XRD). Synthesized AgNPs were quasi-spherical in shape with an average size of 13.09 ± 8.05 nm. 17% EDTA-AgNPs was effective to inhibit *C*. *albicans* and *S*. *aureus* in planktonic and biofilm cultures. The smear layer removal and demineralizing effect were similar between 17% EDTA-AgNPs and 17% EDTA treatments. The 17% EDTA-AgNPs solution proved to be an effective antimicrobial agent, and has a similar chelating capacity to 17% EDTA alone. These *in-vitro* studies strongly suggest that EDTA-AgNPs could be used for effective smear layer removal, having an antimicrobial effect at the same time during RCT.

## Introduction

A successful root canal treatment (RCT) requires the use of chelating agents to achieve effective smear layer removal, which is an amorphous layer from remnants of dentin tissue containing mainly minerals such as calcium [1]. Currently, ethylenediaminetetraacetic acid (EDTA) has been shown to be the most frequently used irrigant solution because of its great ability to form a stable complex with calcium ions, at 15–17% for 1–10 min, as a final rinse [2–5]. However, one of its limitations is the little or null antimicrobial activity over planktonic cells [6–8] and microbial biofilms [9–11] owing to the short application time required for RCT. Therefore, in order to achieve effective root canal disinfection, strong antimicrobial solutions are still needed during the RCT. In recent years, some modifications attempting to overcome this limitation have been evaluated. For instance, alternating sodium hypochlorite with EDTA could promote the disinfection of the root canal [12], but excessive dentin erosion is produced [13]. Recently, a mix of EDTA and the biocide chlorhexidine was introduced; however, a total disinfection has not been achieved [14, 15]. Even more alarming is that bacteria can be resistant or tolerant to chlorhexidine [16], thus the optimal disinfection of the root canal system remains challenging to date.

Therefore, the use of silver nanoparticles (AgNPs) could have many advantages over antimicrobial drugs and disinfectants. First, no specific antimicrobial target mechanism exists; thus, antimicrobial resistance is unlikely. For instance Ag^+^ ions are constantly delivered from the surface of NPs, and their high reactivity triggers elevated intracellular levels of reactive oxygen species (ROS) provoking the disruption of lipids, proteins and DNA, ending in cell death [17]. Second, AgNPs could increase the effectiveness of antimicrobial therapies commonly used. Several studies have reported that AgNPs in combination with some antibiotics have an enhanced antimicrobial effect, even against multi-resistant microorganisms [18, 19].

In order to prevent the aggregation or dissolution of AgNPs, organic ligands have been commonly used as stabilizers [20]. In this study, we employed the ligands of EDTA as a stabilizer agent during the synthesis of AgNPs (EDTA-AgNPs). Previously, Pan *et al*. [21] capped AgNPs with EDTA to enhance the detection of bovine serum albumin. Here, for the first time, we explored the capacity of AgNPs in 17% EDTA as a new modified irrigant solution with an improved antimicrobial property for RCT. They were tested against the fungus *Candida albicans* and the bacteria *Staphylococcus aureus* implied in RCT failures [22–24]; those recalcitrant pathogens are highly-resistant microorganisms due to their biofilm production capacity. Our results demonstrate that 17% EDTA modified with AgNPs has a dual function as an irrigant: simultaneous chelating and antimicrobial capabilities. This could represent a long-term advantage for reducing the cost and/or optimizing RCT time.

## Material and method

### Experimental solutions

17% EDTA was modified with AgNPs (EDTA-AgNPs) to evaluate its use in root canal treatment (patent pending, provisional no. 008558); additionally, a lower concentration of EDTA (0.6%) was also modified with AgNPs in order to determine if the concentration of EDTA affected the size or shape of synthesized nanoparticles and the antimicrobial activity. Both solutions were prepared by chemical reduction using AgNO_3_, EDTA and NaBH_4_ (more details are in patent 008558). All the chemicals used (Sigma-Aldrich) were of analytical reagent grade and used as received. Stock solution of AgNPs was characterized after 24 h of synthesis.

Before assessing smear layer removal and the demineralizing effect, the antimicrobial activity assay was performed in order to use an appropriate concentration of AgNPs in the experimental solution. Two experimental solutions were chosen: 17% EDTA with 16 and 512 μg/ml of AgNPs (EDTA-AgNPs). 17% EDTA (E) was used as a positive control and no treatment (C^-^) as a negative control.

### Characterization of AgNPs

After 24 h of EDTA-AgNPs synthesis, AgNPs were characterized by UV-Vis spectroscopy (PerkinElmer, Lambda 25) in the wavelength range from 350 to 800 nm with a resolution of 1 nm. Samples were examined under high-resolution transmission electron microscopy (HRTEM) (Tecnai F30 operated at 300 keV) to evaluate the distribution, shape and size of AgNPs. At least 500 particles were measured for each solution to characterize the size distribution of particles. Likewise, ζ-potential of AgNPs was measured in triplicate for each solution in a Microtrac Zetatrac (PMX 300).

### Antimicrobial susceptibility testing of EDTA-AgNPs

0.6% and 17% EDTA solutions with AgNPs were tested against standard strains *Staphylococcus aureus* ATCC 25923 and *Candida albicans* ATCC 24433.

### Planktonic cells

The minimum inhibitory concentration (MIC) was determined in accordance with the CLSI guidelines M27-A3 and M07-A9 for broth microdilution assay [25, 26] and Wiegand *et al*. [27]. RPMI 1610 medium (Sigma-Aldrich) buffered to a pH 7.0 with MOPS (Sigma-Aldrich) buffer and Mueller Hinton Broth (Difco) were used as a growth medium for yeast and bacteria, respectively. Briefly, all testing solutions were diluted with an initial inoculum of bacteria (10^8^ CFU/ml) and yeast (10^6^ CFU/ml) to reach final concentrations of AgNPs (0.015 to 128 μg/ml) tested in a final inoculum of 5 x 10^5^ CFU/ml (bacteria) and 5 x 10^2^ (yeast) CFU/ml. Previously, we ensured similar CLSI breakpoints for fluconazole (USP reference standard, Lot: HIL308) using the same *C*. *albicans* strain as a reference [28]. Cultures were incubated at 37°C for 24 hours at 250 rpm (Orbit Environ Shaker). The MIC was defined as the lowest concentration that inhibited visible growth. After the MIC test, minimum bactericide (MBC) and fungicide (MFC) concentrations were determined by transferring 5 μL from all clear MIC tubes onto trypticase soy (TS) and yeast extract peptone dextrose (YPD) agar, respectively, and incubated at 37°C for 24 h. The MBC/MFC was the lowest concentration that killed ≥ 99.9% of cells. The MIC and MBC/MFC were determined in triplicate and were carried out on at least three different days. The MFC/MBC: MIC ratio was used to determine whether the antimicrobial action was microbicidal (MFC/MBC:MIC ≥ 2) or microbiostatic (MFC/MBC:MIC ≥ 4) [29].

### Biofilm cells

The MIC determination on biofilm cells was obtained through the metabolic activity using the XTT [2, 3-bis (2-methyloxy-4-nitro-5-sulfo- phenyl)-2H-tetrazolium-5-carboxanilide] colorimetric assay [30]. *C*. *albicans* and *S*. *aureus* biofilms were developed on the surface of 96-well polystyrene plates adding 1 x 10^6^ and 1 x 10^8^ in RPMI and Mueller Hinton broth, respectively and incubated at 37°C for 24 h. Following incubation, the biofilms were washed with PBS and broth medium containing various concentrations of AgNPs (1–512 μg/ml) was added to the adherent cells, the plates were incubated at 37°C for 1, 10 and 30 min. After the treatment, 100 μl of XTT-menadione solution (1μM) was then added to each prewashed wells and the negative control wells. The plates were incubated in the dark for 2 h at 37°C, and then the absorbance was measured at 490 nm using a microtiter plate reader. The minimum inhibitory concentration was established as the lowest concentration leading to a 50% (MIC_50_), 80% (MIC_80_) and 90% (MIC_90_) reduction in cell viability; this was calculated as follows: O.D. negative control x % MIC / 100. The experimental data were obtained by triplicate in two assays performed on different days.

### SEM evaluations

Both *C*. *albicans* and *S*. *aureus* biofilms were developed on root canal bovine dentin using a CBR 90 CDC Biofilm Reactor (BioSurface Technologies). Before biofilm formation, bovine roots were split to obtain different dentin specimens from the root canal (5 x 5 mm) side and embedded in a polymeric disk (1.2 cm diameter). Each disk was mounted in a coupon holder under sterile conditions. The samples were divided randomly to be inoculated with 1–5 x 10^8^ CFU/ml of *S*. *aureus* and 1 x 10^6^ CFU/ml of *C*. *albicans* and incubated for 16 h at 37 ^o^C (adherence period). After that, the disks were washed with PBS 1x and incubated with new culture media for up to 24 h at 37°C. Five samples were used for each treatment (16 and 512 μg/ml of AgNPs in 17% EDTA) during each time exposition (1,10 and 30 min). Finally, samples were fixed with 2.5% glutaraldehyde and dehydrated in graded ethanol; these were then critical point dried and sputtered with gold (15 s at 40 mA). An ESEM (Environmental Scanning Electron Microscopy, FEI Quanta FEG 250) was used to evaluate microbial biofilms; cell diameter measurements were carried out randomly in each sample/treatment (n = 150).

### Chelating activity of EDTA-AgNPs

#### Sample preparation

Dentin fragments were prepared as follows: one hundred seventeen fragments (2.5 x 2.5 mm) were obtained from along the root canal dentin portion of freshly extracted non-carious bovine teeth. All fragments were immersed in 5.25% NaOCl in order to produce organic tissue dissolution. The fragments were divided randomly into two independent assays: 1) for smear layer removal and 2) for demineralizing effect. For each assay the samples were split into two subgroups for exposure time (1 and 10 minutes) and for the different treatments (n = 7).

#### Smear layer removal

The root canal dentin side of each fragment was ground for 5 s at a designated constant speed using #600 SiC paper discs to produce a flat surface with a smear layer. After this procedure, samples were immersed in 1 ml of experimental solutions for 1 or 10 min. After treatments, the flattened side of samples was examined by AFM (Nano-surf Easy Scan 2, SPM Electronics, Liestal, Switzerland) in contact mode with a silicon nitride (SiN) probe at a scanning rate of 49.5 μg/s. The surface roughness (*R*_*a*_) and depth profile (*R*_*v*_) were evaluated at the same scan size (49.5 x 49.5 μm^2^) in triplicate in different areas. Furthermore, a negative control group of samples (n = 7) was analyzed before treatments. After AFM evaluation, representative samples of each group (n = 3) were scanned under ESEM at 500x and 2,500x. Dentin surfaces treated with experimental solutions were analyzed for the presence of AgNPs by backscattered electron detector (BSE) and energy dispersive spectroscopy (EDS).

To evaluate dentin macroscopic changes, the same dentin surface of three randomly selected specimens was examined under a stereoscopic microscope (Leica EZ4HD) with a similar set of parameters, before and after treatments. It is important to mention that based on the antimicrobial assays, smear layer removal and further analysis were carried out with freshly prepared EDTA-AgNPs solutions.

#### Demineralizing effect

Samples were prepared as previously described for smear layer removal. Thereafter, dental fragments were immersed in 10 ml of the corresponding treatment solution under stirring at 1 and 10 min. Afterwards, the fragments were removed and the remaining solution was used for calcium (Ca) and magnesium (Mg) quantification, as main basic metal constituents of mineralized dentin [31]. The quantification of Ca and Mg in each solution was measured by atomic absorption spectroscopy (AAS) (AAanalyst 400, Perkin Elmer LCC) using air-acetylene flame at wavelength of 414 nm for calcium and 285 nm for magnesium. Experimental solutions in absence of specimens were used as blanks. To maximize the accuracy of readings, all treatment solutions were diluted in a 0.2% lanthanum solution.

Finally, the samples used to determine Ca and Mg were evaluated in a microhardness testing machine (Snowon, HVS-100, China) and a diffractometer (Rigaku SmartLab, Cu K*α*, 44 mA current, 40 Kv voltage) to estimate the changes on the dentin structure. The indentations (n = 5) for the microhardness test (MH) were made at randomly selected areas in each specimen under a 200 g load and 15 s dwell time. To record the X-ray diffraction (XRD) patterns of dentin hydroxyapatite (HAp), the treated samples were reduced to dentin powder and thereafter examined from 20^o^ to 70^o^ at a scanning rate of 2.83^o^ /min.

#### Statistical analysis

To determine significant differences between treatments, the Kruskal-Wallis and post hoc Dunn’s tests were applied, followed by the Mann-Whitney U test for pairwise comparison between exposure times (SPSS v.15.0). Significance was established at *P* < 0.05.

## Results

### Characterization of AgNPs

**Fig 1:**
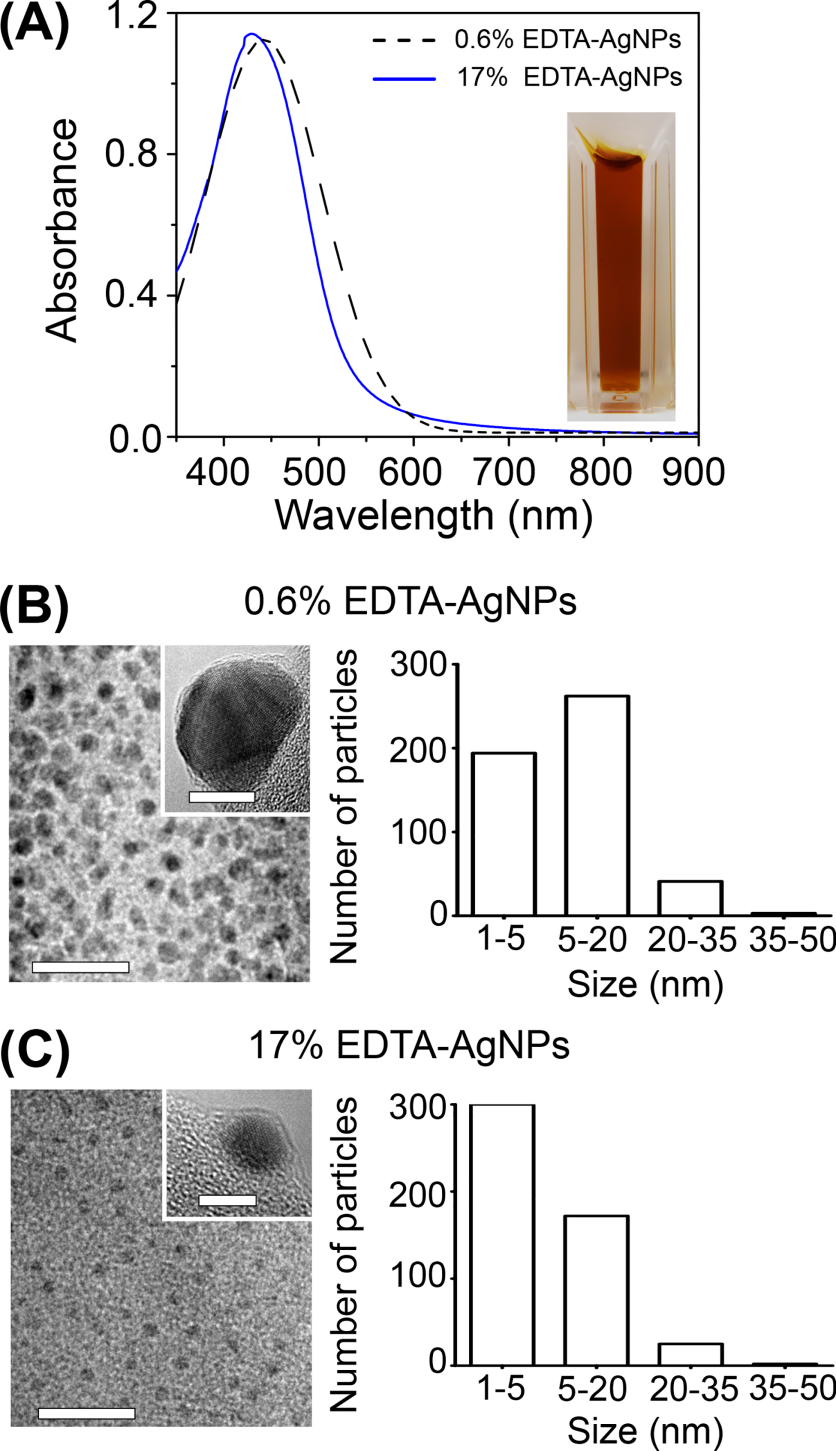
Characterization of AgNPs synthesized in EDTA. (A) Uv-vis spectra, (B-C) TEM micrographs with size distribution histogram (n = 500) of AgNPs synthesized in 0.6% (B) and 17% EDTA (C). Scale bars: (B-C) 20 nm, inset (B) 10 nm, and inset (C) 5 nm.

A pale yellow to light-brown coloration was observed immediately after synthesis of AgNPs in 0.6% and 17% EDTA solutions (Fig 1A). The presence of AgNPs was confirmed for each solution by the UV-vis analysis; an intense absorption band appeared at 430–440 nm (Fig 1B). To corroborate shape and size of particles, samples were analyzed under TEM and the presence of small quasi-spherical AgNPs was revealed (Fig 1B and 1C, inset). Particles synthesized in both EDTA concentrations had similar size ranges (5–50 nm) (Fig 1B and 1C); however, at 0.6% EDTA the average size was 15.80 ± 7.41 nm, whereas the average size of AgNPs in 17% EDTA was slightly smaller (13.09 ± 8.05 nm) with a higher number of smaller particles (5 nm or less) (Fig 1C). The mean value of ζ-potential for AgNPs in 0.6% EDTA was -32.93 ± 7.66 mV and for AgNPs in 17% EDTA it was -31.30 ± 6.86 mV.

### Antimicrobial activity of AgNPs in planktonic and biofilm cells

In general, AgNPs in 17% EDTA were highly active against *S*. *aureus* and *C*. *albicans*. For planktonic cells, the MIC recorded for AgNPs in 17% EDTA was at least 8-fold less compared to 0.6% EDTA. Similarly for microbial biofilm, the lowest MICs recorded were at 17% EDTA-AgNPs for *C*. *albicans* during 1–30 min, while for *S*. *aureus* the lowest MICs values were at 0.6% EDTA-AgNPs from the first minute.

**Fig 2:**
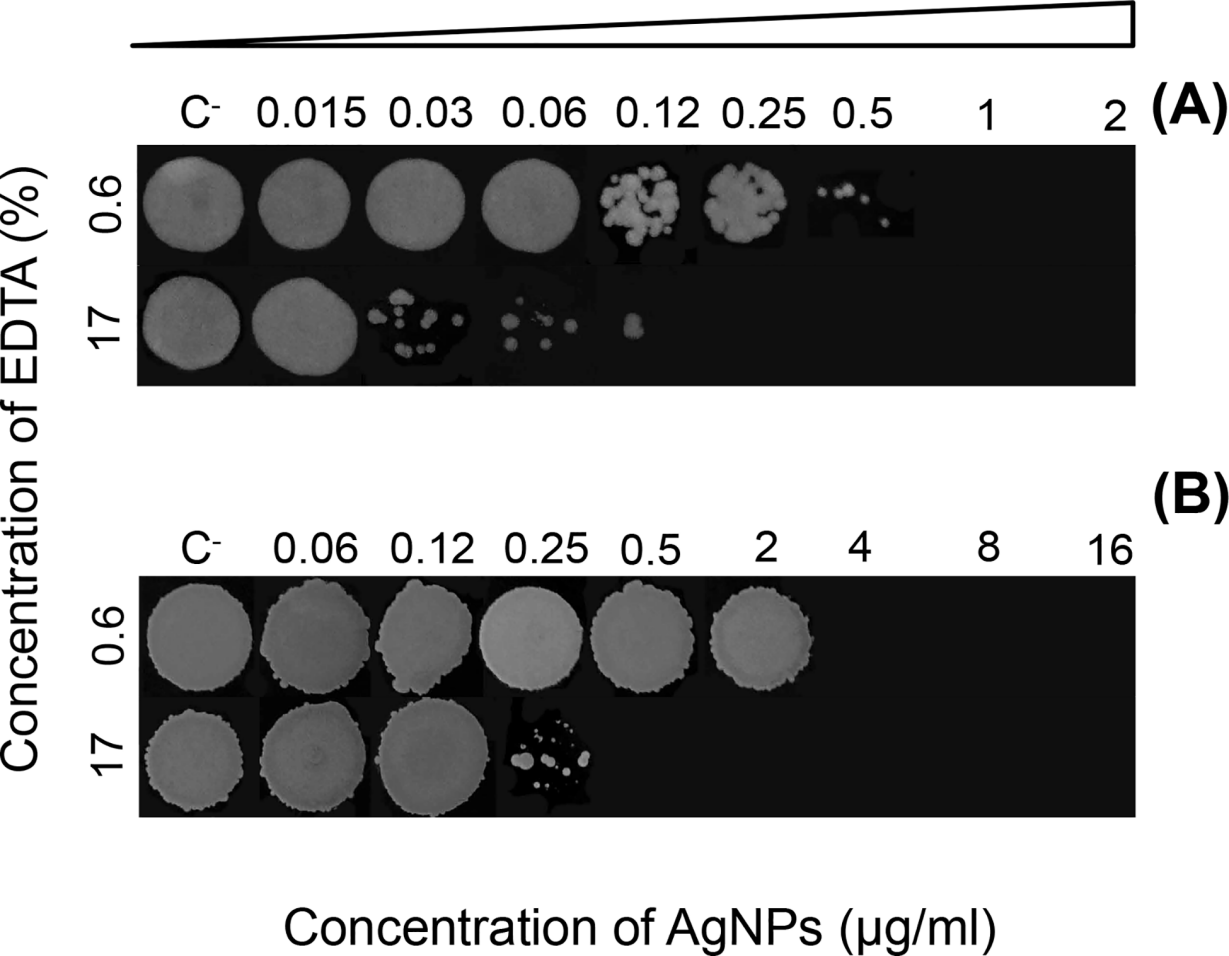
**Growth of (A) *C*. *albicans* and (B) *S*. *aureus* colonies treated with AgNPs in 0.6% and 17% EDTA for MFC/MBC determination after broth microdilution assay.** C^-^ = negative control.

Regarding the MBC and MFC in planktonic cells, AgNPs in 17% EDTA for *S*. *aureus* and *C*. *albicans* were 8-fold and 4-fold less, respectively, compared to those obtained at 0.6% EDTA (Table 1). Yet, the MFC:MIC ratio was 4-fold higher for AgNPs at 17% EDTA *C*. *albicans*; the number of colonies decreased in a dose-dependent manner at low concentrations compared to 0.6% EDTA (Fig 2).

**Table 1:** MIC and MFC/MBC values for microbial planktonic cells treated with AgNPs (μg/ml) in 0.6% and 17% EDTA.

**Solutions**	***C*. *albicans***		***S*. *aureus***	
	MIC	MFC	MIC	MBC
**17% EDTA**	35.3	70.6	83	> 300
**0.6% EDTA-AgNPs**	0.5	1	4	4
**17% EDTA-AgNPs**	0.062	0.25	0.25	0.5

**Fig 3:**
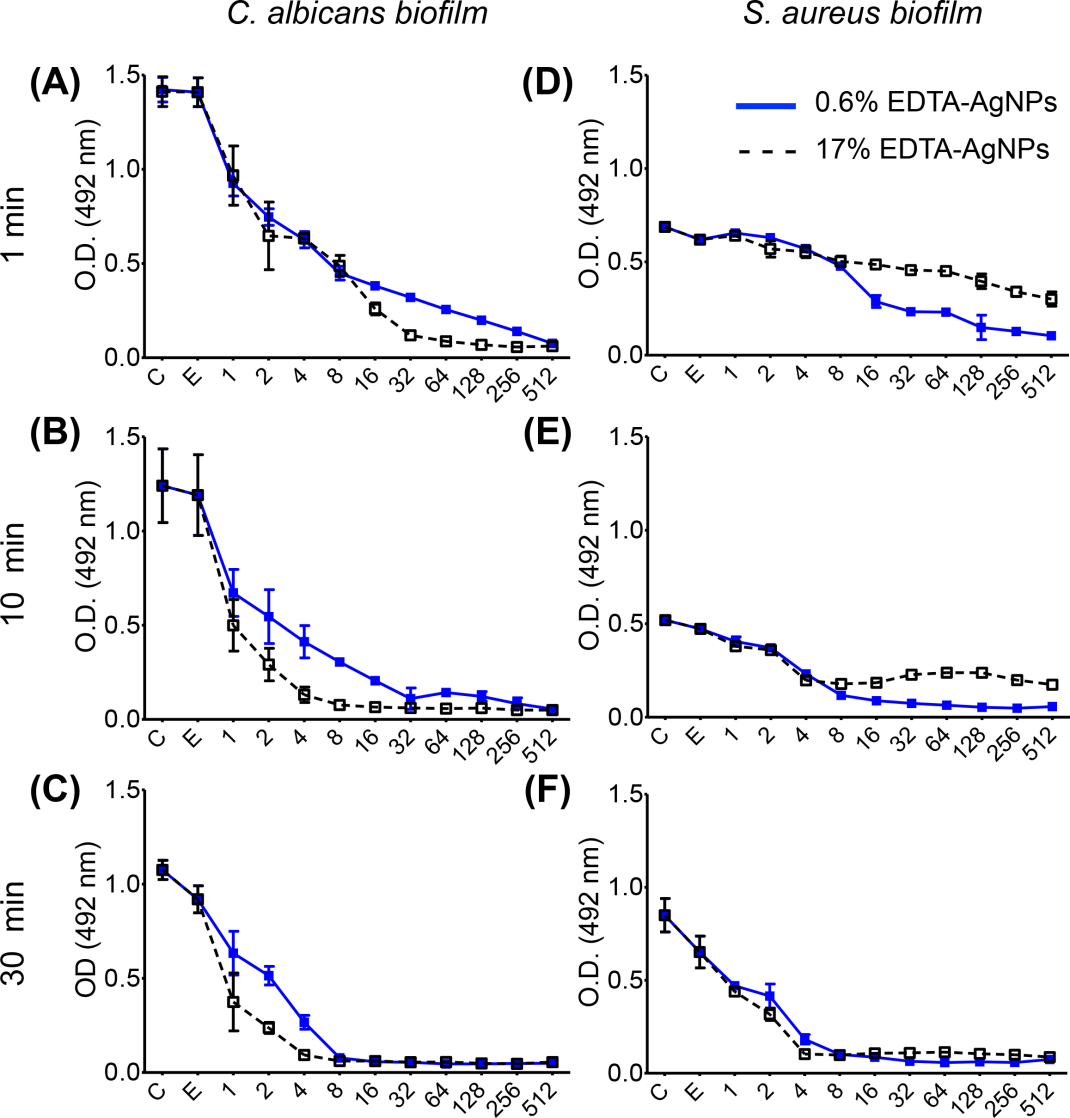
**Metabolic activity of (A-C) *C*. *albicans* and (D-F) *S*. *aureus* biofilm treated with AgNPs in 0.6% and 17% of EDTA at several concentrations (1–512 μg/ml) at 1 (A,D), 10 (B,E) and 30 (C,F) min.** C = negative control, E = 17% EDTA.

In the XTT assays, the viability of cultures exposed only to EDTA showed a slight decrease in comparison with the negative control for both microorganisms, being more noteworthy over 1 min (Fig 3). On the other hand, the biofilm viability of both *C*. *albicans* and *S*. *aureus* exposed to 17% and 0.6% EDTA-AgNPs (1 to 512 μg/ml) dropped significantly at all concentrations of AgNPs compared with 17% EDTA alone (*p* <0.05). Comparing the concentration of EDTA, a synergic effect was observed against *C*. *albicans* at 17%, while this trend was opposed for *S*. *aureus* from 8 to 512 μg/ml. As a result, 90% of *C*. *albicans* biofilms were affected when they were exposed to 17% EDTA-AgNPs at 1–30 min (Table 2). In contrast, only 50% of *S*. *aureus* biofilms exposed to 17% EDTA-AgNPs were non-viable during 1 (MIC_50_: 128 μg/ml) and 10 (MIC_50_: 4 μg/ml) min, followed by a reduction of 90% in 30 min (Table 3).

**Table 2:** MIC values of AgNPs (1–512 μg/ml) in 0.6% and 17% EDTA solutions against *C*. *albicans* biofilm.

Solutions	1 min		10 min		30 min	
	MIC_50_	MIC_90_	MIC_50_	MIC_90_	MIC_50_	MIC_90_
**0.6% EDTA-AgNPs**	4	256	2	32	2	8
**17% EDTA-AgNPs**	2	32	1	4	1	4

**Table 3:** MIC values of AgNPs (1–512 μg/ml) in 0.6% and 17% EDTA solutions against *S*. *aureus* biofilm.

Solutions	1 min		10 min		30 min	
	MIC_50_	MIC_90_	MIC_50_	MIC_90_	MIC_50_	MIC_90_
**0.6% EDTA-AgNPs**	16	>512	4	256	2	32
**17% EDTA-AgNPs**	128	>512	4	>512	2	512

**Fig 4:**
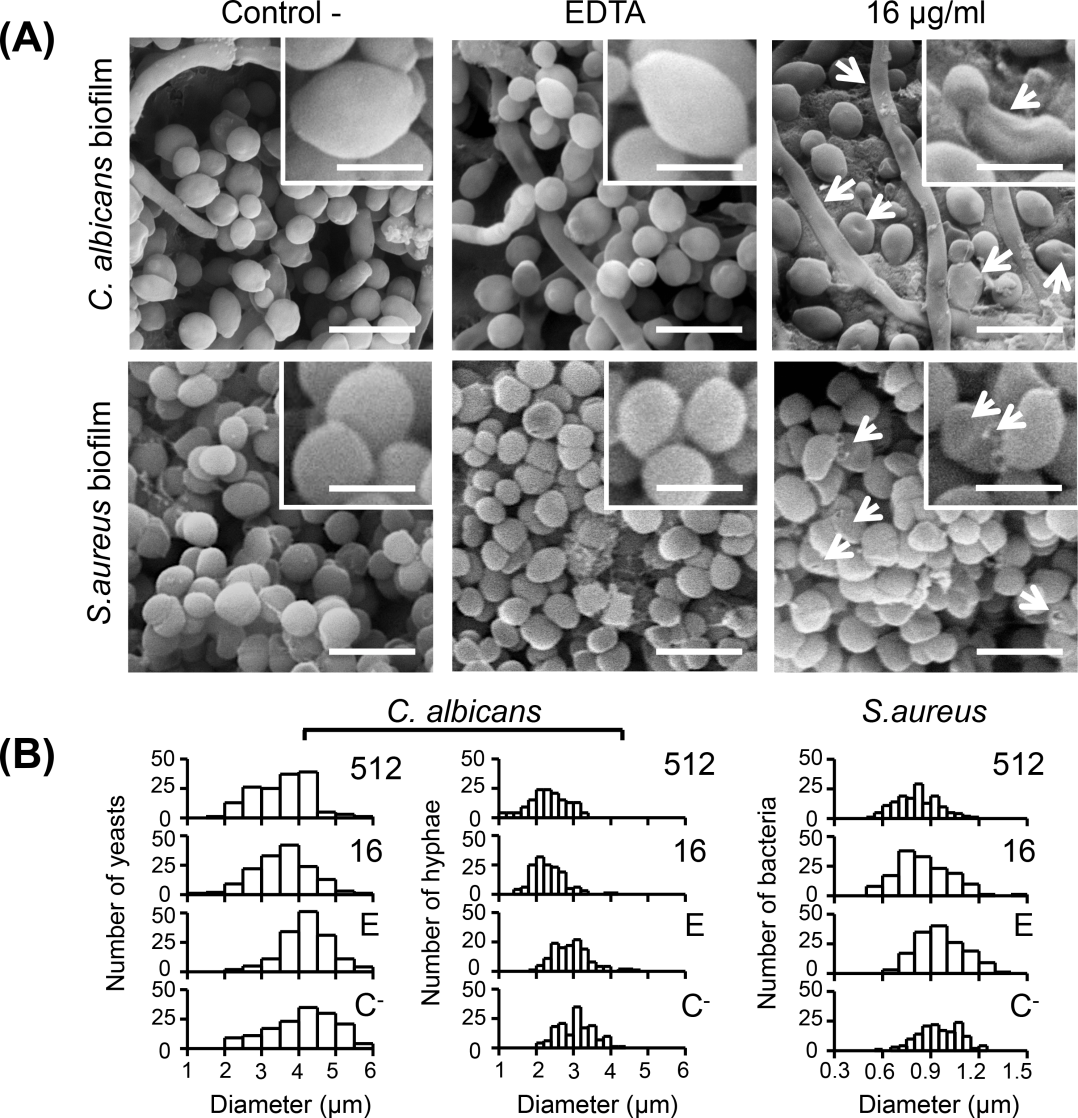
**SEM micrographs (A) and diameter distribution histograms (B) of *C*. *albicans* and *S*. *aureus* cells in biofilms exposed to PBS 1x as control, 17% EDTA and AgNPs (16 μg/ml) in 17% EDTA during 10 min**. Loss of cell volume and pits are pointed out (arrows, insets). Scale bars: (*C*. *albicans*) 10 μm and (*S*. *aureus*) 2.5 μm.

Biofilms of *C*. *albicans* and *S*. *aureus* exposed to AgNPs in 17% EDTA were examined under SEM in order to observe the effect of this solution on the external morphology of both microorganisms. Normal cell morphology was observed in cells not exposed to the experimental treatments; those exposed only to EDTA apparently were not affected in their external structure (Fig 4A and 4B). However, morphological changes indicative of cell damage were observed in cells exposed to treatments for 10 min, even at the lower concentration of AgNPs (16 μg/ml) (Fig 4). Cells of *C*. *albicans* and *S*. *aureus* exposed to EDTA-AgNPs were observed with loss of cell volume appearance, which increased the population with decreased cell diameter (Fig 4A and 4B); also some cells were observed with pits (Fig 4A, arrows).

**Fig 5:**
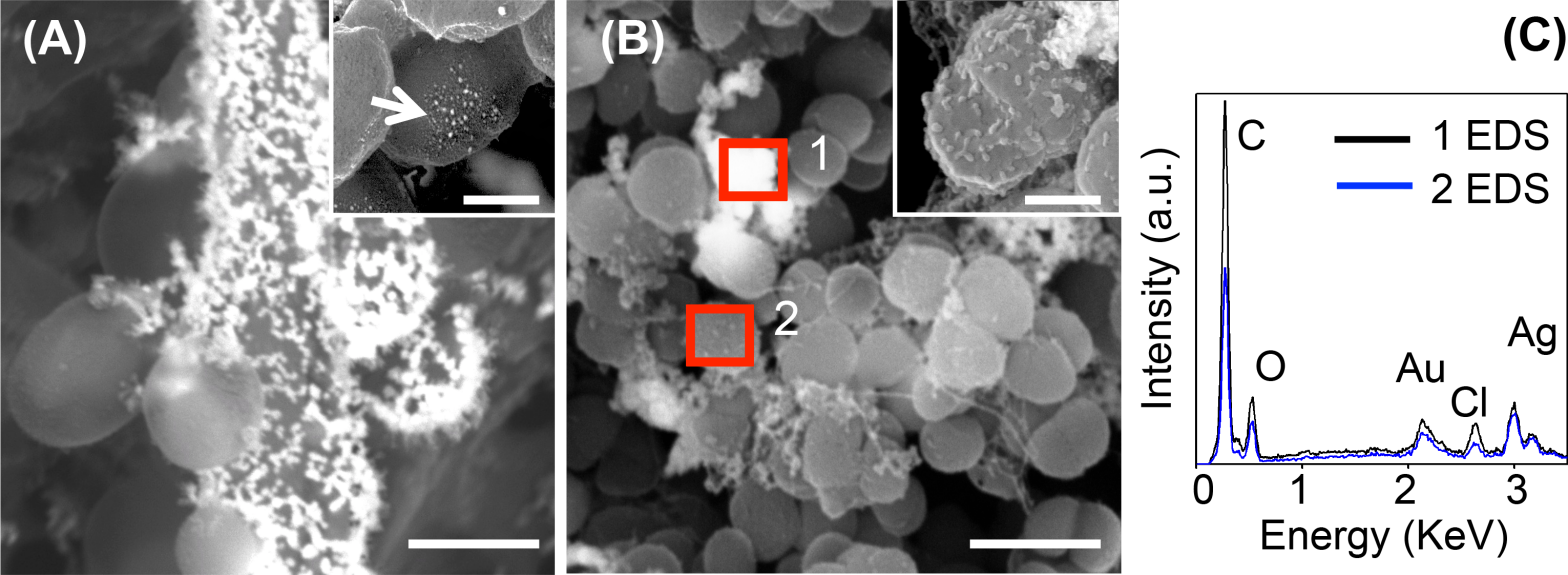
SEM micrographs of *C*. *albicans* and *S*. *aureus* biofilm exposed to AgNPs (512 μg/ml) in 17% EDTA after 10 min. (A) yeasts and hypha of *C*. *albicans* covered with silver agglomerates, inset showing the presence of small agglomerates in yeasts; (B) cells of *S*. *aureus* completely covered with AgNPs (enclosed area 1) and others with smaller agglomerations (enclosed area 2), inset showing cell deformation; (C) EDS analysis providing the chemical composition of enclosed area 1 (1 EDS) and enclosed area 2 (2 EDS) over the cell surface of *S*. *aureus*. Scale bars: A = 5 μm (inset = 1 μm); B = 2 μm (inset 0.5 μm).

At the highest concentration of EDTA-AgNPs tested (512 μg/ml), the presence of AgNPs agglomerates were seen over both microbial cells (Fig 5). In *C*. *albicans* agglomerations were more evident in hyphae (Fig 5A), while in yeasts smaller agglomerations were observed (Fig 5A inset). In *S*. *aureus* the presence of agglomerates was also evident (Fig 5B) and cell shrinking were commonly observed (Fig 5B inset). The chemical analysis (Fig 5C) corroborate that AgNPs are completely covering some cells (Fig 5B, enclosed area 1) and are present as spots over the majority of cells (Fig 5B, enclosed area 2).

### Smear layer removal

**Fig 6:**
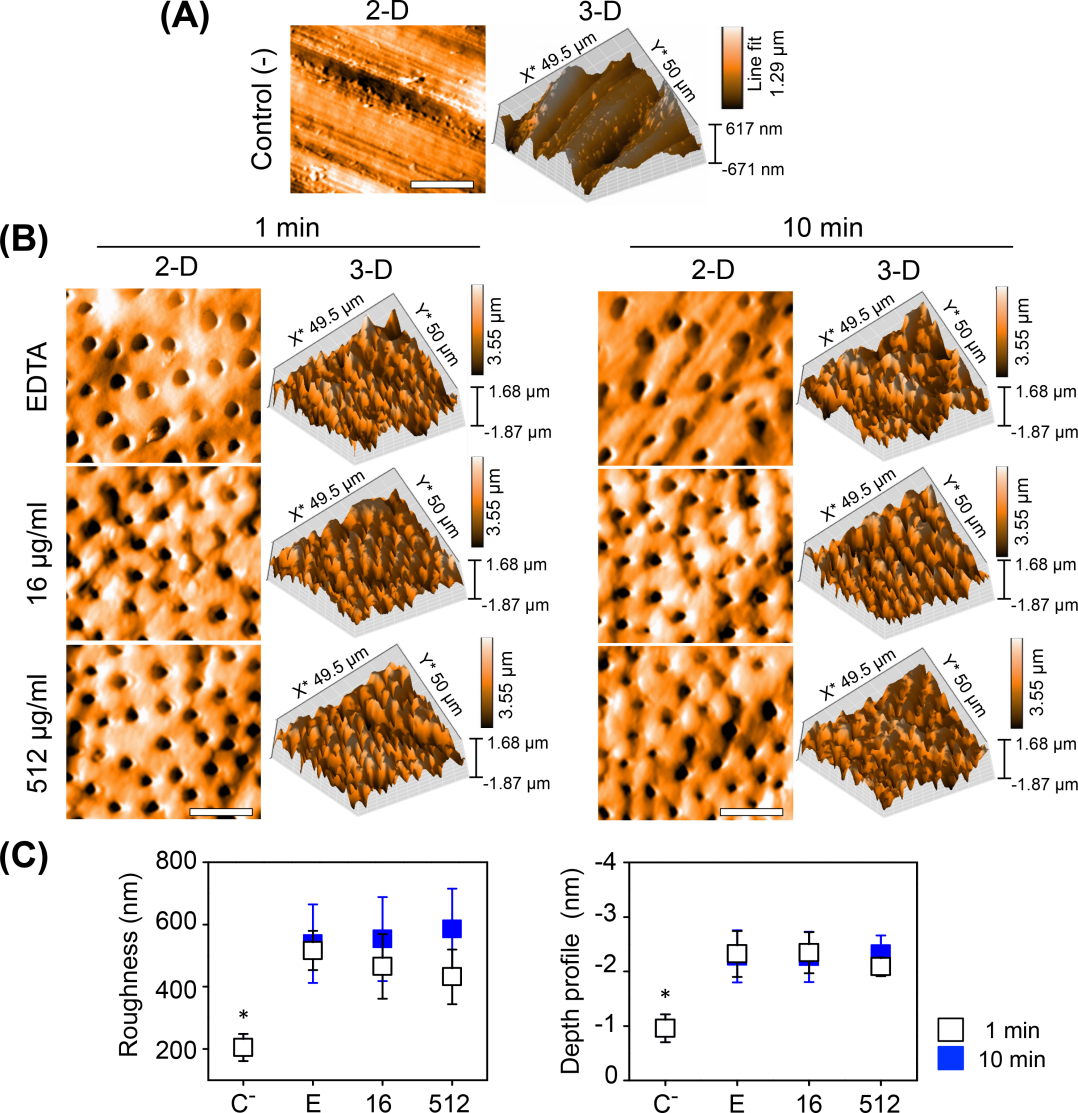
AFM analysis of dentin treated for smear layer removal. (A-B) 2-D and 3-D AFM images, (C) surface roughness and mean depth values of the smear layer removal in treated dentin after 1 and 10 min of exposure time. Negative control (C^-^), 17% EDTA (E) and two concentrations of AgNPs (16 and 512 μg/ml) synthesized in 17% EDTA. (*) Kruskal Wallis Test with post hoc Dunn's test (*P* <0.05). Scale bars, 20 μm.

Surfaces of treated and non-treated dentin samples were analyzed by AFM, images show topographic differences between them. In non-treated samples, 2-D and 3-D AFM images show the smear layer completely covering dentin tubules (Fig 6A), whereas dentin tubules were fully open in samples exposed to the EDTA-AgNPs solutions (Fig 6B). The analysis indicate that the smear layer was removed from the dentin surface, and also from inside the dentinal tubules at both concentrations, and at each exposure time (Fig 6B). These observations were corroborated by the surface roughness and depth profile values which were statistically different (*P* < 0.05) between treated and non-treated samples. No significant differences between treatments were found (Fig 6C). For exposure times, AFM images and values also revealed that there was not a marked difference in a time-dependent manner after removing the smear layer.

**Fig 7:**
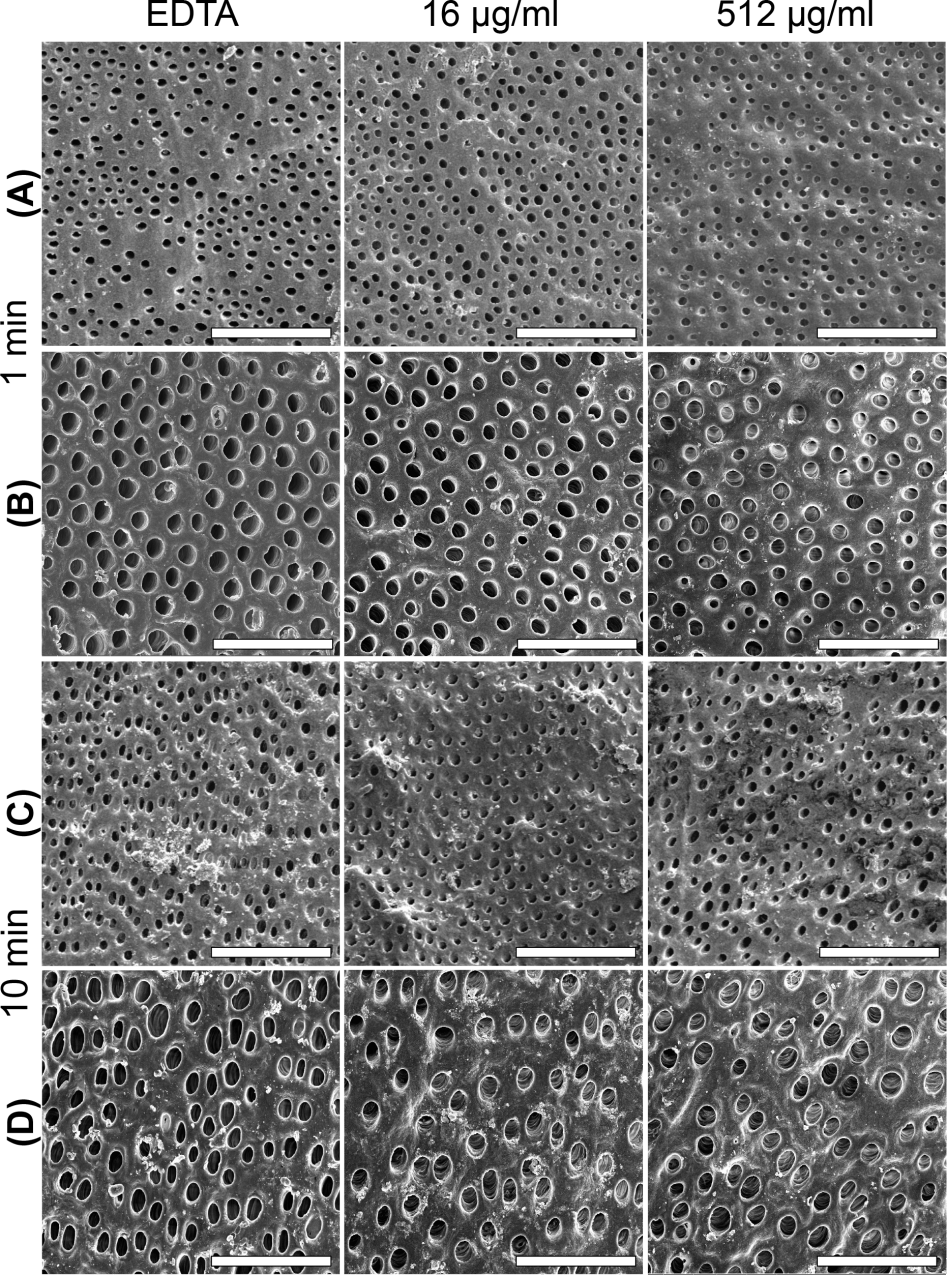
SEM micrographs of dentin treated with 17% EDTA and 17% EDTA-AgNPs. Low (A, C) and high (B, D) magnifications show dentin free of smear layer at all treatments tested at 1 and 10 min. Scale bars: (A, C) 100 μm; (B, D) 20 μm.

**Fig 8:**
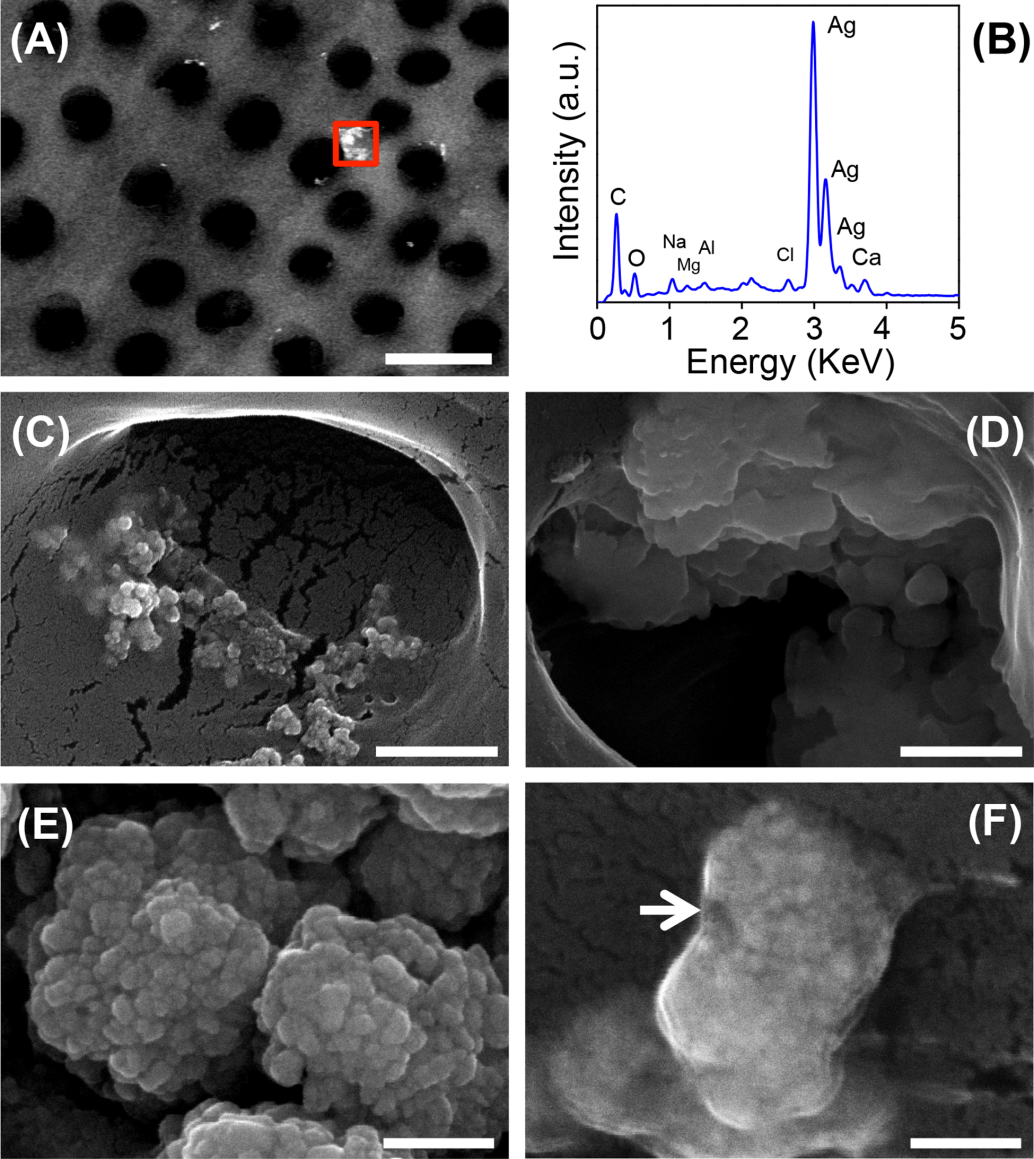
SEM micrographs of dentin treated with 17% EDTA-AgNPs at 512 μg/ml of silver concentration. (A) BSE provided the localization of non-uniform aggregates, (B) EDS spectrum showing the presence of silver in the aggregate shown in (A), (C) silver aggregates seen outside and (D) inside dentinal tubules, (E) higher magnification of aggregates, (F) some aggregates were observed probably covered by EDTA (arrow). Scale bars: (A) 5 μm, (C-D) 500 nm, (E-F) 200 nm.

AFM observations were further supported by SEM analysis that exhibits the apertures of dentinal tubules opened and free of smear layer (Fig 7). However, SEM examination at low (Fig 7A and 7C) and high (Fig 7B and 7D) magnifications revealed that surfaces treated for 10 min show some dentinal tubules with a slightly wider diameter compared to those treated for 1 min. Concerning the localization of AgNPs in samples treated with the higher concentration of AgNPs, they were clearly seen along treated surfaces by the BSE detector (Fig 8A), and corroborated by the Ag signal in the EDS analysis (Fig 8B). At higher magnifications, AgNPs were visible outside (Fig 8C) and inside (Fig 8D) dentinal tubules, some of them forming aggregates with particles of 40 nm in diameter (Fig 8E), and some others most likely covered by EDTA (Fig 8F).

**Fig 9:**
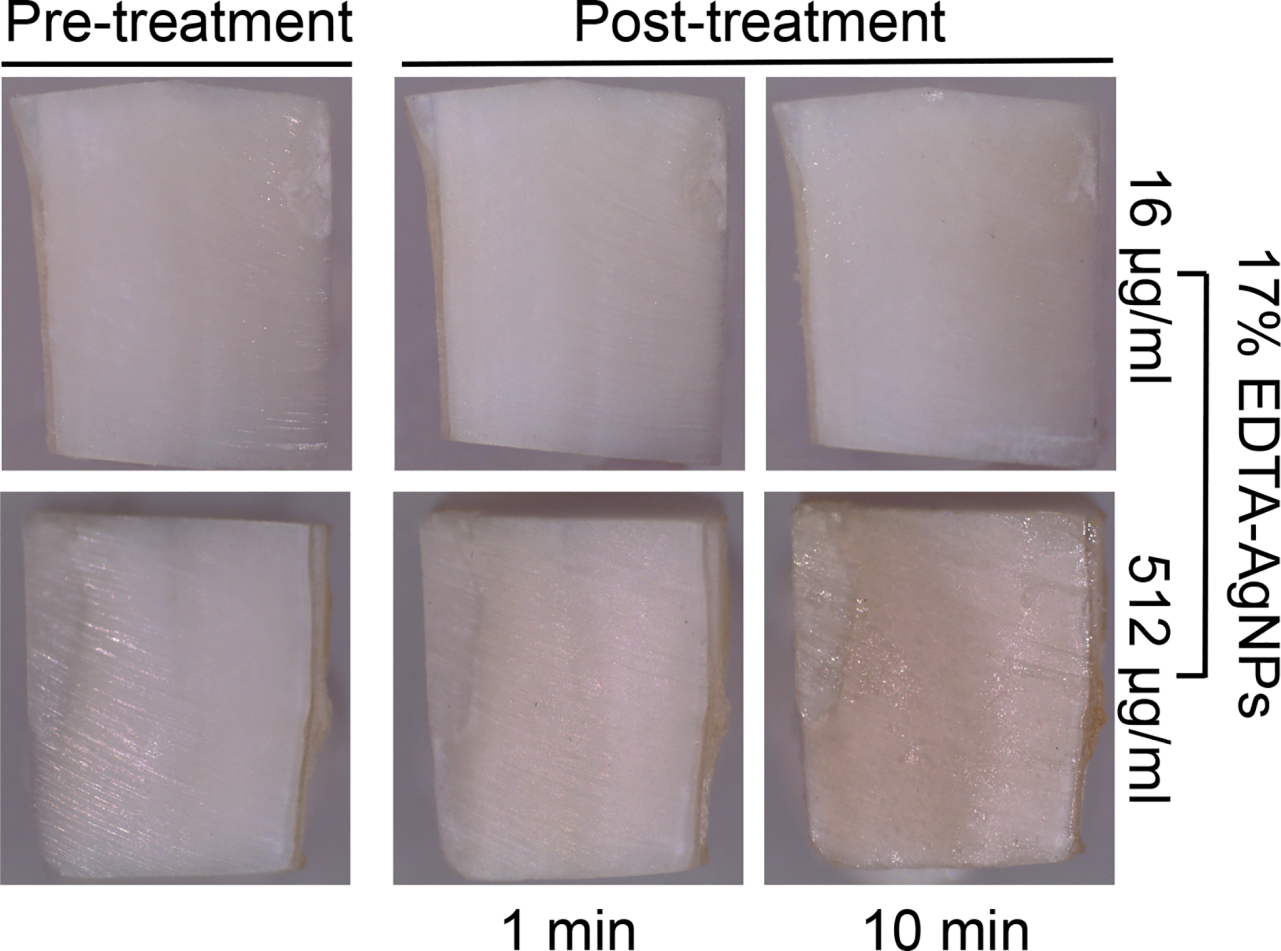
Stereoscopic images of dentin treated with 17% EDTA-AgNPs at two silver concentrations after 1 and 10 min.

Macroscopically, the dentin surface exposed to the EDTA-AgNPs solutions showed a slight darkening color, which was more evident at the higher concentration of AgNPs at 10 minutes of treatment (Fig 9).

### Demineralizing effect

**Fig 10:**
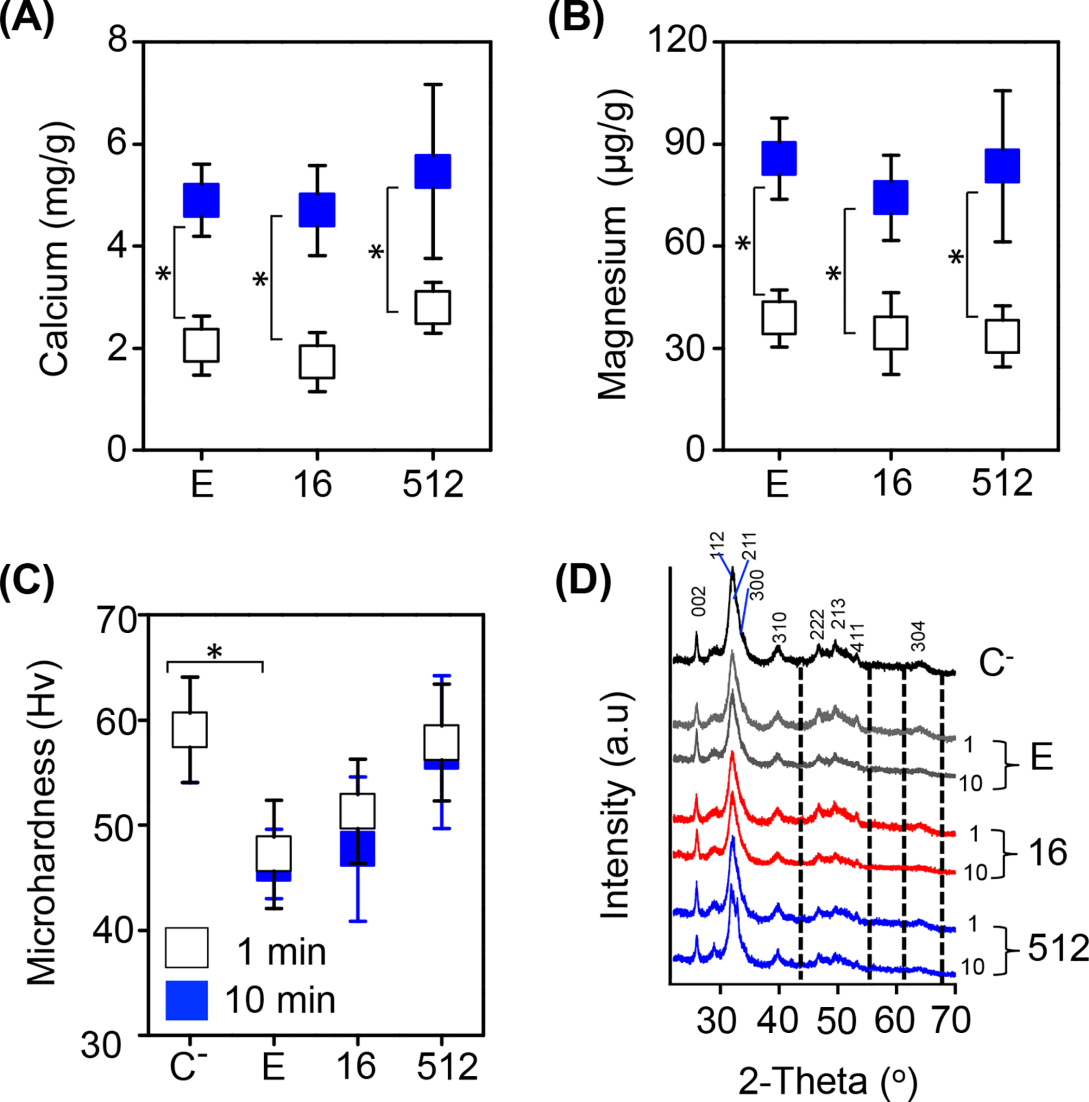
Demineralization values of dentin treated with 17% EDTA-AgNPs after 1 and 10 min. Average and standard deviation of (A) amount of extracted calcium, (B) extracted magnesium, (C) MH values and (D) XRD patterns. Negative control (C^-^), 17% EDTA (E) and two concentrations of AgNPs (16 and 512 μg/ml) synthesized in 17% EDTA. (*) Mann-Whitney U test and Kruskal Wallis Test with post hoc Dunn's test (P < 0.05).

Fig 10 shows the results of Ca/ Mg (Fig 10A and 10B), MH values (Fig 10C) and XRD patterns of treated dentin (Fig 10D) with each irrigant solution and immersion time. No significant difference was found for the amount of extracted metal ions between each irrigant solution (*P* > 0.05), whereas these increased significantly (*P* < 0.05) after 10 min of immersion. For dentin MH, the extracted Ca/ Mg were only reflected in a significant decrease (*P* < 0.05) over the surface MH of dentin treated with EDTA without AgNPs, compared to the control group (59.05 ± 5.02 Hv). Interestingly, MH values increased in treated specimens with all concentrations of AgNPs. XRD patterns of dentin indicated that only the HAp intensity at 122 and 211 peak decrease from the first minute while the rest (222, 213, 411 and 304) decreased slightly after 10 min of treatment with 17% EDTA and 17% EDTA-AgNPs. There was no difference between each irrigant.

## Discussion

A new plausible modification of 17% EDTA with AgNPs is proposed for root canal treatment. 17% EDTA is one of the most commonly used irrigants for smear layer removal due to its excellent chelating capacity [2–5]; however at the short time of application it has almost null antimicrobial properties [6–11]. Therefore, through the addition of AgNPs we obtained a modified EDTA solution with increased antimicrobial capabilities without loosening its chelating ability.

**Fig 11:**
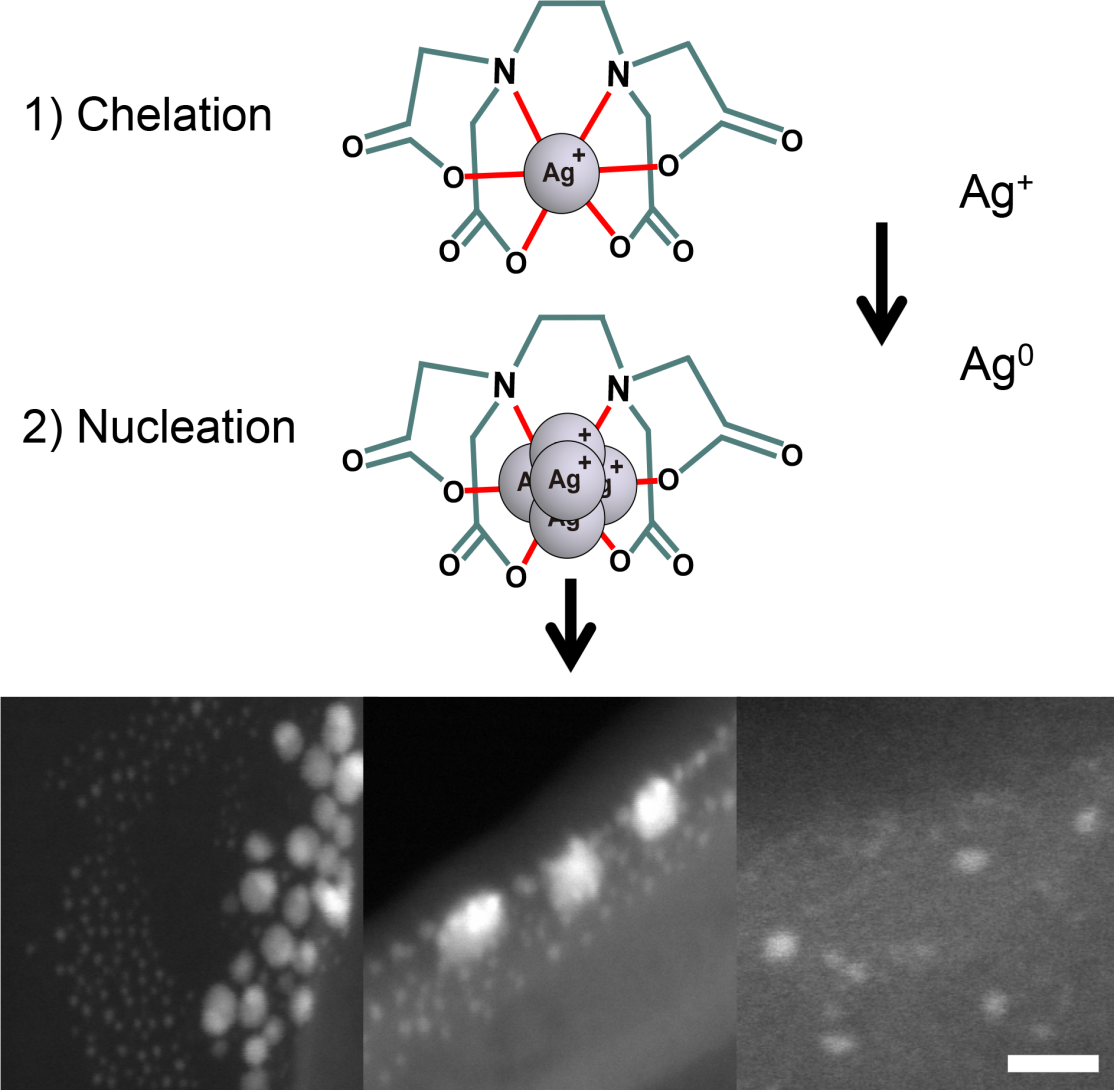
Plausible role of EDTA in the synthesis of AgNPs. Scale bar: 5 nm.

The formation of AgNPs in 17% EDTA was successfully achieved, and to elucidate how this concentration may influence the development of particles and thus affect their antimicrobial capacity, a lower concentration of EDTA (0.6%) was used for AgNPs synthesis. Stock solutions of AgNPs were analyzed for stabilization by a zeta potential analyzer, and shape and size distribution by Uv-Vis spectrophotometry and TEM. In general, the shapes of synthesized AgNPs were quasi-spherical, absorption peaks at 17% and 0.6% EDTA were indicative of spherical AgNPs [32]; at 0.6% it slightly shifted towards the red wavelengths indicating somewhat bigger particles [33]. These observations were corroborated with the average particle size distribution obtained by TEM examination. Results of ζ-potential indicate that at least within the first 24 h, AgNPs in both solutions were stabilized by EDTA since values higher than 30 mV to -30 mV predict stable AgNPs [34]. However, AgNPs synthesized in 17% EDTA were partially dissolved compared to 0.6% EDTA stock after 3 months of storage. Concerning this, it has been reported that EDTA can stabilize AgNPs through an electrostatic double layer, providing a repulsive force to silver colloid [20, 21]. In accordance with these previous studies, our results strongly suggest that EDTA may participate as a stabilizer agent, controlling the size of particles during the nucleation process; due to Ag^1+^ ions being attached to negative moieties in EDTA. This could be true even for particles less than 1 nm in diameter, as we can observe under HR-TEM on STEM mode for synthesis in 17% EDTA after 24 h (Fig 11). On the other hand, the stability of AgNPs can be compromised by oxidative dissolution that increases as the EDTA concentration increases with lower concentration of AgNPs (S1 Fig), other factors that may affect stability are a decreasing pH, or the quantity of O_2_ presented in the solution, affecting the smaller AgNPs more rapidly [35–37]. Despite this, Ag_total_ concentration remained constant in the EDTA-AgNPs solutions for at least 5 days without precipitating, as determined by AAS (S2 Fig).

The antimicrobial susceptibility assays showed that AgNPs in EDTA confers antimicrobial activity against *C*. *albicans* and *S*. *aureus*. Nevertheless at 17% EDTA-AgNPs, the higher MBC (MFC) / MIC ratio obtained for *C*. *albicans* planktonic cells and the delayed bactericidal effect for *S*. *aureus* biofilm, suggest that some Ag^+^ ions were complexed at more concentration of EDTA, mitigating the toxicity of AgNPs but without affecting the overall antimicrobial capacity, as reported for various ligands [38]. Despite this, the MICs and MFC/MBC for planktonic cells and MICs for *C*. *albicans* biofilm required for microbial inhibition were surprisingly much less with AgNPs in 17% EDTA (Tables 1 and 2). Thereafter, Live/dead assays were carried out on microbial biofilms grown onto root canal dentin, however the results were not reported because EDTA itself affects the cell permeability by chelating Ca^2+^ and Mg^2+^ in the Gram-negative lipopolysaccharides layer and Gram-positive peptidoglycan layer [39, 40] and could give false-positive staining for propidium iodide dye. Nevertheless, the treated samples were used to observe the ultrastructural changes in the microbial biofilm in which cell wall collapse was more noteworthy for *C*. *albicans* than *S*. *aureus* (Fig 4). Furthermore, higher accumulations of AgNPs were detected on the surface of hyphae, compared with yeast or bacterial cells (Fig 5). This supports the synergic toxicity of 17% EDTA-AgNPs over *C*. *albicans* biofilm (Table 2), very likely due to the sequestering of calcium essential for cell turgidity [41] in combination with the highly reactive Ag^+^ ions. For instance, when EDTA is combined with some antimicrobial agents, it enhances bactericidal [40, 42, 43] and fungicidal activity [44, 45]. Here, although EDTA is chelating Ag^+^ ions, we believe that the antimicrobial activity of 17% EDTA was enhanced with the addition of AgNPs in various ways: it allows direct penetration of free Ag^+^ ions to the cytoplasmic membrane; additionally, the high concentration of EDTA dissolves AgNPs, increasing the release of Ag^+^ ions, and consequently less concentration of AgNPs was needed for antimicrobial effect. On the other hand, if more Ag^+^ ions are chelated, it is most likely that less antimicrobial/chelating activity will be obtained; thus, long storage time could affect the effectiveness of the solution.

Once the antimicrobial capacity of EDTA-AgNPs was corroborated, the chelating capacity was evaluated indirectly (smear layer removal) and directly (demineralizing effect) in order to determine if the chelating effectiveness was altered by the presence of AgNPs. Thus, the performance of freshly-prepared EDTA-AgNPs was evaluated at two concentrations of AgNPs (16 and 512 μg/ml) during short and prolonged time intervals (1 and 10 min) as reported in other works [46].

As for the exposure time of dentin to EDTA or EDTA-AgNPs, AAS results revealed a more remarkable difference between exposure times due to the demineralizing effect, which barely affected the crystallinity of HAp. However, we did not observe a clear relationship between those observations and MH results in a time-dependent manner; this was most likely because after treatments it was necessary to apply an extra polish to the samples in order to produce valid indentations for MH measurements, and this could influence final values. Furthermore, the different results between assessments were probably related to the volume used for the demineralizing effect (10 ml) and smear layer removal (1 ml), which has been related to the aggressiveness of EDTA when used at up to 1 ml for RCT [13]. In SEM examinations an increased number of opened dentin tubules, some of them with wider lumen, were clearly visualized at 10 min. Whereas, AFM roughness values revealed only slightly difference at 10 min, not statistically different from 1 min. There was also no difference in the depth value between treatments; this is probably because it depends on the high tip; therefore, it is difficult to approach real values considering that EDTA has shown to produce demineralization up to a depth of 40 μm [4]. Clearly, for this type of samples SEM analysis is suitable to complement AFM results, such as the time of micrograph acquisition, and the possibility of closer examinations of dentin ultrastructure at higher magnifications.

According to the overall results, AgNPs did not change the chelating capacity of 17% EDTA at low and high concentration of AgNPs at 1 and 10 mins of treatment. The non-uniform aggregates of AgNPs at the higher concentration (512 ug/ml), seen over and inside the dentin tissue were related to the slight darkening color of the dentin tissue, which at lower concentrations of AgNPs is negligible (Fig 9). In addition to this, the dentin had an increased MH value, even at low concentrations of AgNPs (16 μg/ml). All these findings were corroborated in a competence assay between EDTA and murexide chelators (S3 Fig) where the concentrations of AgNPs tested were not high enough to saturate the EDTA capacity for calcium chelation. In other words, if a higher number of EDTA molecules are available, the chelation of silver ions in a minimum amount will not affect the smear layer removal. Furthermore, because Ag^+^ ions have lower stability constants (Log *K* = 6–7) to form complexes, the high selectivity of EDTA for Ca^2+^ and Mg^2+^ was another beneficial factor during complexation. Even this affinity could participate in the dispersion and distribution state of AgNPs [47]. Clearly, for smear layer removal 17% EDTA-AgNPs could represent a potential clinical alternative against resistant microbes from dental root canal infections. Moreover, AgNPs concentrations below 50 μg/ml are reported to have no toxicity effect for endodontic purposes, Gomes-Filho et al (2010) reported that 23 μg/ml appears to be non toxic and 47 μg/ml had similar tissue reaction to 2.5% sodium hypochlorite [48]. Additionally, this novel modification may have a prolonged antimicrobial effect as well as reinforcing the demineralized dentin.

## Conclusions

It was found that EDTA plays an important role in the size and stabilization of AgNPs; it allows the formation of small AgNPs and they are stable for at least 24h, in a silver concentration dependent manner. According to the *in-vitro* analysis, 17% EDTA-AgNPs showed antimicrobial activity against planktonic cells and microbial biofilms. Furthermore, it was found that it has similar chelating capacity to 17% EDTA at 1 and 10 min of treatment at low and high concentration of AgNPs (16 and 512 μg/ml).

## Supporting information

S1 Fig **UV-Vis spectra of EDTA-AgNPs solutions at 16 (A-B) and 512 (C-D) μg/ml of silver concentration in 0.6% (A-C) and 17% (B-D) EDTA.** The solutions with 512 μg/ml of AgNPs were assessed from 0, 0.5, 1, 4, 6 and 24 h. For 16 μg/ml of AgNPs solutions, the UV-vis spectra were taken each 3 minutes until the yellow color was not seen by the naked eye (0.5 h). For both AgNPs concentrations, 0 h was considered as the first moment of AgNO_3_ reduction by NaBH_4_.
(TIF)

S2 Fig Total concentration of Ag from stocks of 16 (unfilled squares) and 512 (filled squares) μg/ml of AgNPs in 17% EDTA. The figure shows the mean and standard deviation of 3 lectures from the first day of AgNPs synthesis (0 day), 1 and 5 days.
(TIF)

S3 Fig Uv-Vis spectra of the chelation competition assay for Ca^2+^ between murexide and EDTA-AgNPs solutions. Both chelators were used 1:1 and exposed with 2400 μg/ml of calcium to visualize Ca^2+^-complex reaction with murexide. EDTA was adjusted to equal concentration of murexide since it was used as a saturated solution (1% w/v). Murexide with (black line) and without (black dashed line) calcium; Murexide-EDTA exposed to calcium: red line and Murexide-EDTA with a final AgNPs concentration of 16 (blue line) and 512 (blue dashed line) μg/ml exposed to calcium.
(TIF)

S1 File Database. (XLSX)
