# Supplementary figures and images for: Dual function of EDTA with silver nanoparticles for root canal treatment–A novel modification
Source: PLoS One. 2018 Jan 18;13(1):e0190866. doi: 10.1371/journal.pone.0190866 (PMC5773103; doi:10.1371/journal.pone.0190866)

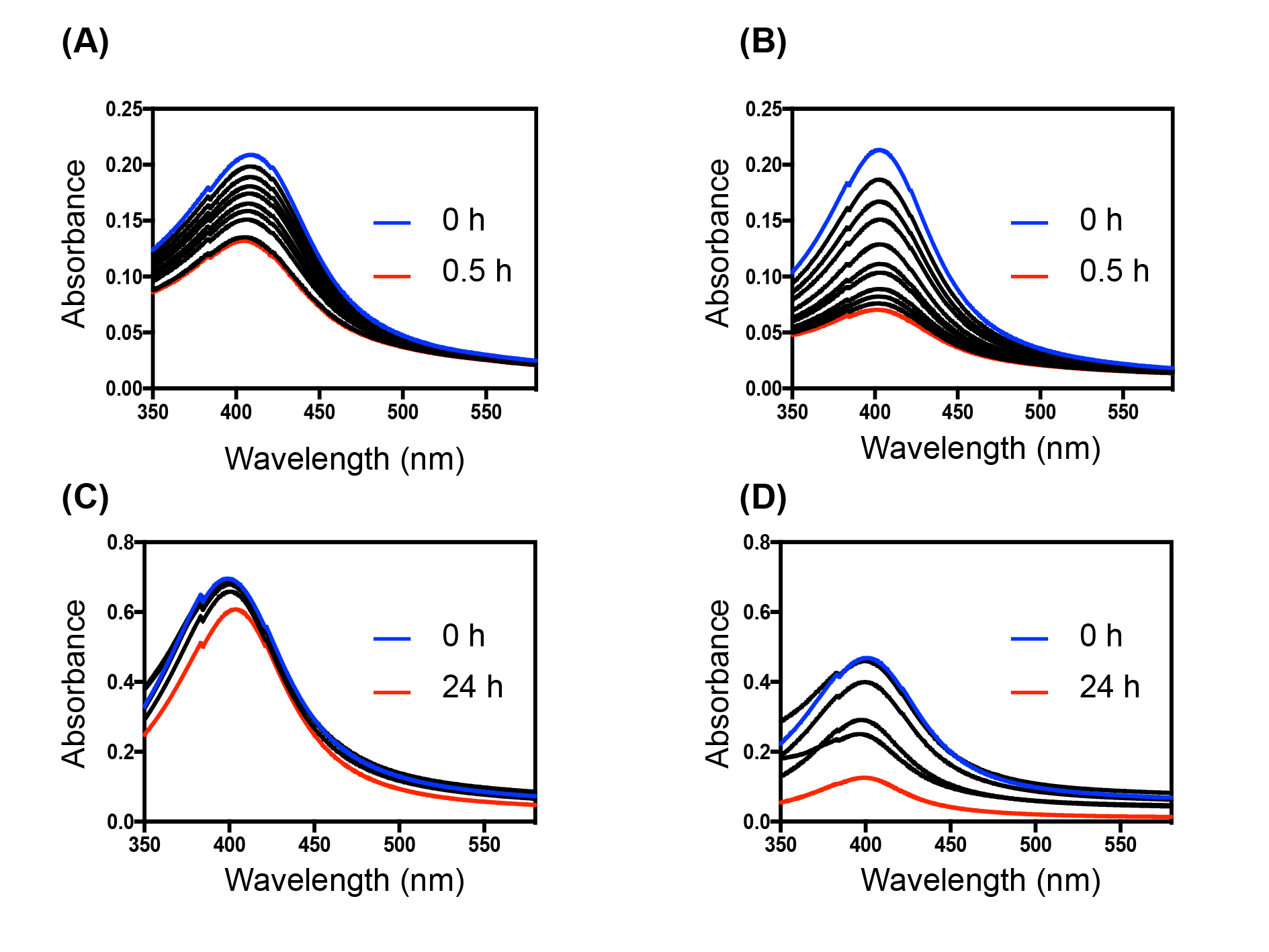

Supplement: S1 Fig — UV-Vis spectra of EDTA-AgNPs solutions at 16 (A-B) and 512 (C-D) μg/ml of silver concentration in 0.6% (A-C) and 17% (B-D) EDTA. The solutions with 512 μg/ml of AgNPs were assessed from 0, 0.5, 1, 4, 6 and 24 h. For 16 μg/ml of AgNPs solutions, the UV-vis spectra were taken each 3 minutes until the yellow color was not seen by the naked eye (0.5 h). For both AgNPs concentrations, 0 h was considered as the first moment of AgNO3 reduction by NaBH4. (TIF) [file pone.0190866.s001.tif]

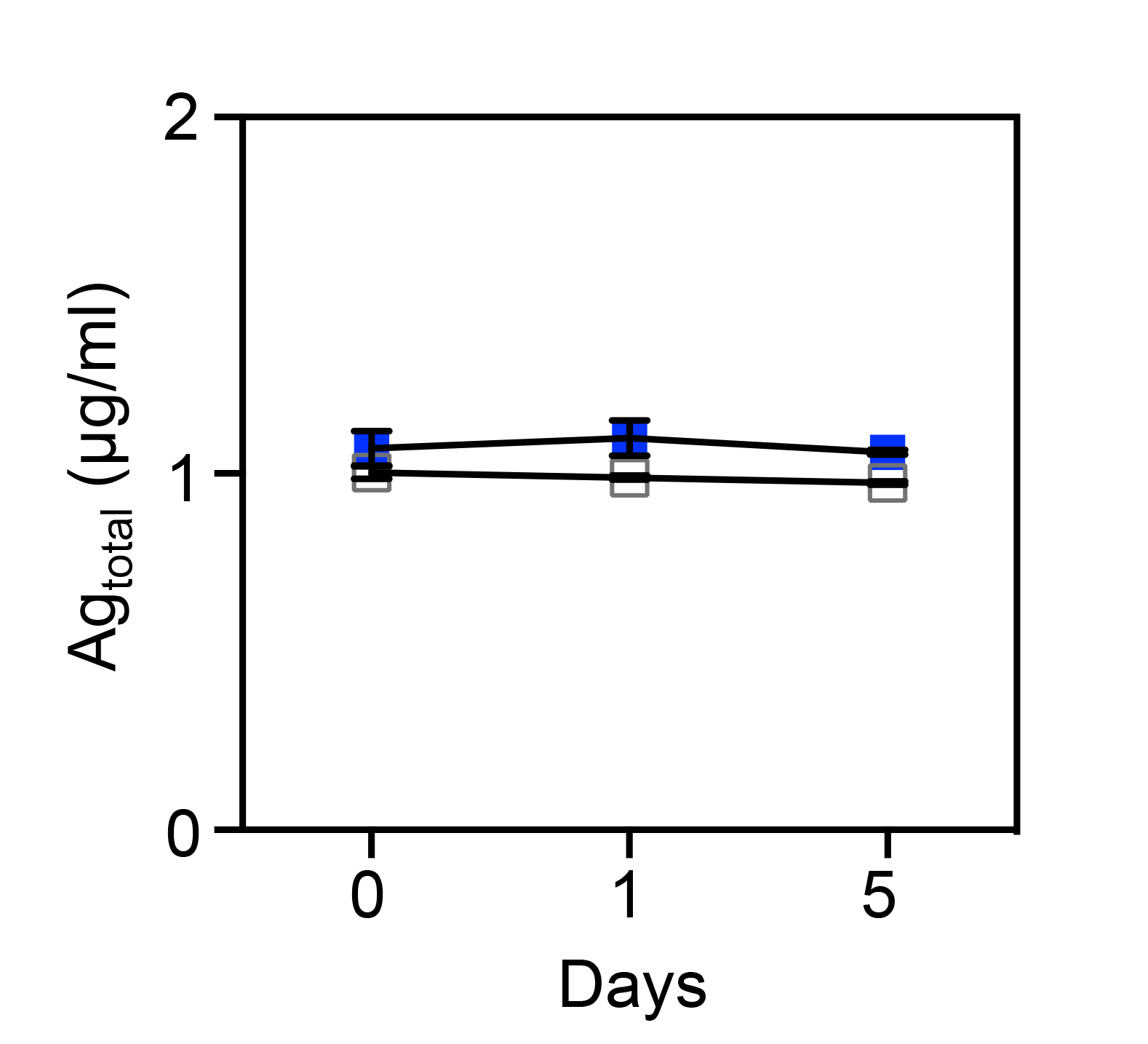

Supplement: S2 Fig — The figure shows the mean and standard deviation of 3 lectures from the first day of AgNPs synthesis (0 day), 1 and 5 days. (TIF) [file pone.0190866.s002.tif]

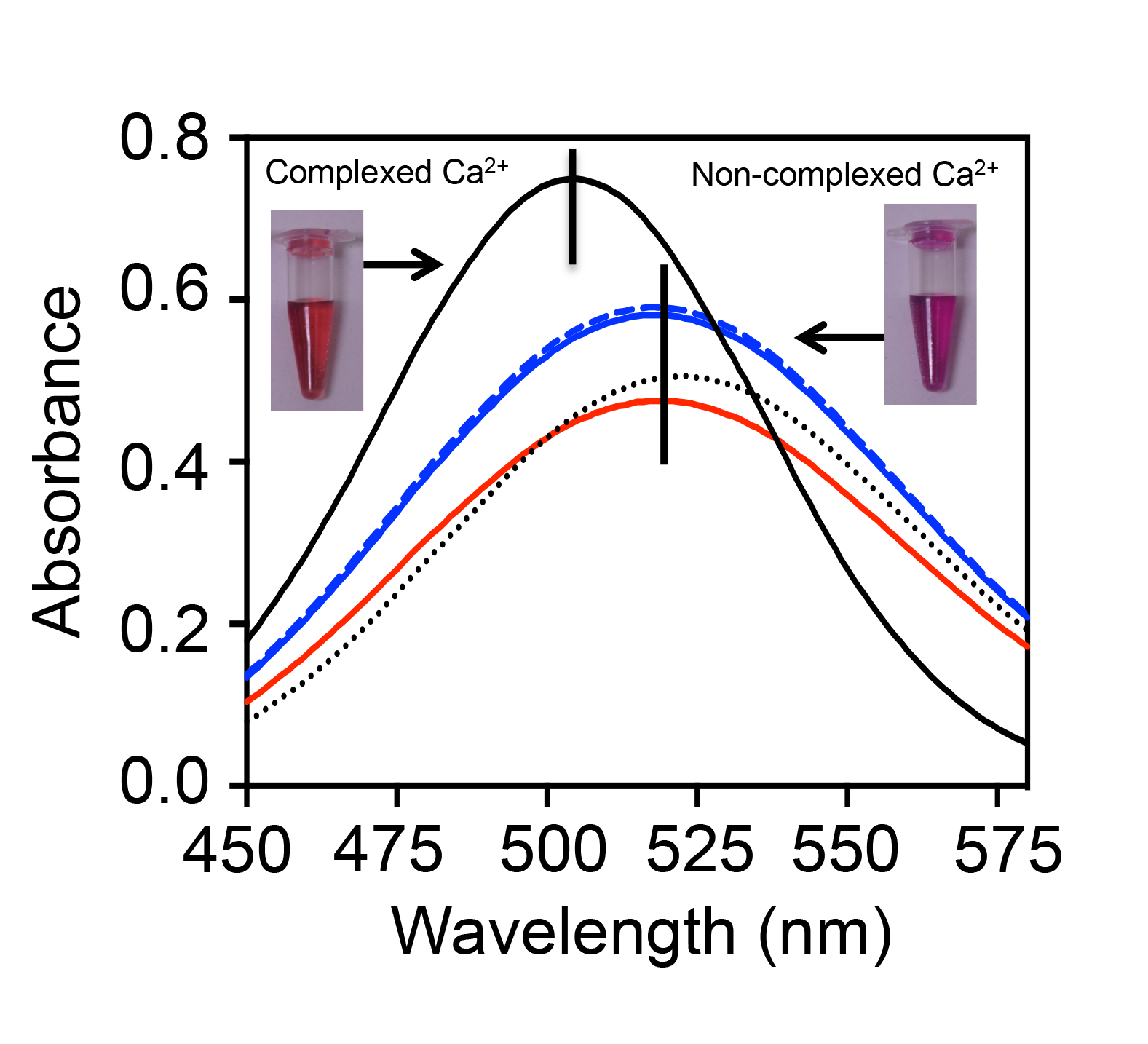

Supplement: S3 Fig — Both chelators were used 1:1 and exposed with 2400 μg/ml of calcium to visualize Ca2+-complex reaction with murexide. EDTA was adjusted to equal concentration of murexide since it was used as a saturated solution (1% w/v). Murexide with (black line) and without (black dashed line) calcium; Murexide-EDTA exposed to calcium: red line and Murexide-EDTA with a final AgNPs concentration of 16 (blue line) and 512 (blue dashed line) μg/ml exposed to calcium. (TIF) [file pone.0190866.s003.tif]
